# SCREENING CUTOFF VALUES TO IDENTIFY THE RISK OF FALLS AFTER STROKE: A SCOPING REVIEW

**DOI:** 10.2340/jrm.v56.40560

**Published:** 2024-10-24

**Authors:** Daisuke MATSUMOTO, Takaaki FUJITA, Ryuichi KASAHARA, Kenji TSUCHIYA, Kazuaki IOKAWA

**Affiliations:** 1 Department of Occupational Therapy, School of Health Sciences, Fukushima Medical University, Fukushima; 2 Department of Rehabilitation, Kita-Fukushima Medical Center, Date; 3 Faculty of Health Sciences, Nagano University of Health and Medicine, Nagano, Japan

**Keywords:** Berg Balance Scale, Falls Efficacy Scale-International, fall risk, stroke, Timed Up and Go test

## Abstract

**Objective:**

The present scoping review aimed to summarize and determine the accuracy of the variables and cutoff values reported to date for identifying fall risk in patients with stroke and identify the commonalities, limitations, and clinical implications.

**Methods:**

Articles published by the end of 2023 were searched using PubMed, Cumulative Index of Nursing and Allied Health Literature, and Scopus electronic databases. Two reviewers created a search formula, searched the databases, and conducted primary and secondary screenings.

**Results:**

This review included 21 articles. The most commonly used individual indicator for identifying fall risk after stroke was the Berg Balance Scale; the cutoff values were relatively consistent, ranging between 46.5 and 50.5 points (area under the curve: 0.72–0.81). For the Timed Up and Go test and Falls Efficacy Scale-International, the cutoff values were in the range of 15–19 s and 27–29 points, respectively, and were relatively consistent across the articles. However, the area under the curve values were low (0.66–0.70 and 0.68–0.71, respectively).

**Conclusion:**

Among various assessments, the Berg Balance Scale is the most extensively studied tool, with established cutoff values associated with falls risk. It serves as a reliable indicator for detecting fall risk, especially in community-dwelling individuals with chronic stroke.

## LAY ABSTRACT

Falls are common in people with stroke and often lead to injury, fear of falling, and limitation of daily activities. Effective fall prevention requires prognostication to identify high-risk individuals. The present review summarized the accuracy of rehabilitation-related assessments and cutoff values reported to date for identifying fall risk in patients with stroke. The results showed that cutoff values of 46.5 to 50.5 points on the Berg Balance Scale was a relatively good predictor of falls in community-dwelling individuals with chronic stroke. Cutoff values for other assessments varied widely between previous studies or had insufficient predictive accuracy. We also found that few studies calculated a cutoff value to identify fall risk using cognitive function as an indicator. In conclusion, this review identifies certain limits of accuracy and contributes to the appropriate use of cutoff values in clinical practice.

Stroke is the second leading cause of disability and death worldwide, posing a huge burden at both individual and societal levels (1). According to international guidelines for fall prevention, stroke is one of the common medical conditions associated with a higher fall risk, warranting increase supervision when there are more impairments following a stroke (2). In particular, impaired balance, poor lower limb motor function, visuospatial hemineglect, and a history of falls are associated with a high risk of falling (3, 4). Approximately half of patients with stroke experience at least 1 fall in the first year after a stroke, and falls are 7 times more prevalent in this population than among healthy individuals (5). Thus, after a stroke, patients have a significantly higher risk of fracture (6, 7), with falls often leading to limitations in activities of daily living (8). Furthermore, the fear of falling is reportedly associated with a reduced quality of life (9). Therefore, it is critical to prevent falls in such patients.

The number of prevalent cases of stroke worldwide is huge, exceeding 100 million (10). Therefore, it may not be feasible to provide fall prevention interventions to all stroke patients, and effective fall prevention requires prognostication to identify high-risk individuals. A previous review on fall risk prediction in patients with stroke has indicated that there are several multivariable risk prediction models (11). However, while predictive models with many variables and complex algorithms tend to be more accurate, they are less likely to be widely used in clinical practice (12). Therefore, a predictive model should be simple, with a single-variable cutoff value, for clinical feasibility. A review by Lee et al. (13) of fall screening assessments for older community-dwelling individuals and inpatients reported that 12.3 s is a useful cutoff value for the Timed Up and Go (TUG) test. However, to the best of our knowledge, no review has summarized the cutoff values for predicting falls in patients after stroke. A scoping review is suitable for providing overviews of a broad research field and identifying knowledge gaps. Therefore, the purpose of this scoping review was to (*i*) summarize and determine the accuracy of the variables and cutoff values reported to date that can be used to identify fall risk in patients with stroke, and (*ii*) identify the commonalities, limitations, and clinical implications of the cutoff values.

## METHODS

We established a search formula, searched databases, and conducted primary and secondary screenings based on the Preferred Reporting Items for Systematic Reviews and Meta-Analysis extension for Scoping Reviews (PRISMA-ScR) (14). The eligibility criteria were as follows: (*i*) the participants had experienced a stroke; (*ii*) the study calculated a cutoff value to identify fall risk; (*iii*) the data included the area under the curve (AUC), indicating that accuracy, sensitivity, and specificity were calculated; and (*iv*) studies published up to 31 December 2023. The exclusion criteria were as follows: (*i*) study of falls during acute hospital stay, (*ii*) review articles, (*iii*) conference proceedings, and (*iv*) papers written in languages other than English. The first exclusion criterion was established because the situation during acute hospitalization differs significantly from the subacute and chronic phases, as movement is severely restricted under medical supervision.

The article search was conducted on 3 January 2024, using PubMed, Cumulative Index of Nursing and Allied Health Literature (CINAHL), and Scopus electronic databases. The search formula was developed in consultation with the authors and occupational therapists (DM and TF): ([stroke] OR [cerebrovascular diseases] OR [CVA]) AND ([falls] OR [accidental fall]) AND ([cut off] OR [cutoff]). Articles extracted from the databases were imported into EndNote X7 (Clarivate PLC, Philadelphia, PA, USA), and duplicates were removed. For the primary screening, 2 reviewers (DM and TF) independently checked the titles and abstracts. Subsequently, they conducted a full-text search to determine the articles to include based on the eligibility criteria (secondary screening). Disagreements during the primary and secondary screenings were resolved through discussion.

The 2 reviewers independently extracted the following data for integration from the accepted articles: first author name, publication year, country/region, stage of stroke recovery (acute, subacute, chronic), main inclusion criteria, sample size, duration of the fall survey, environmental indicators and their cutoff values, AUC, sensitivity, and specificity. For practicality, data with cutoff sensitivities and specificities of ≥ 50% were primarily integrated.

## RESULTS

### Study selection

**Fig. 1:**
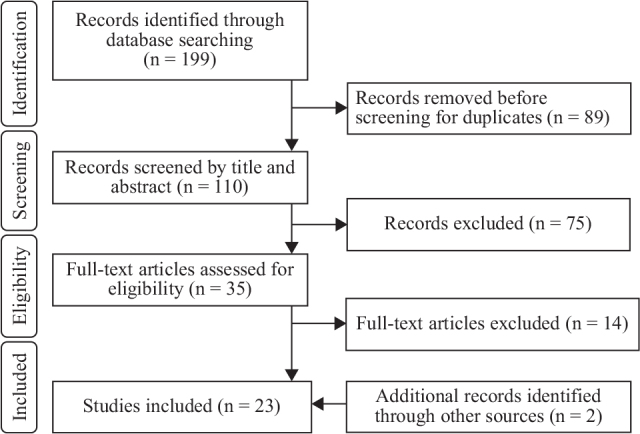
PRISMA flow diagram. PRISMA: Preferred Reporting Items for Systematic Reviews and Meta-Analysis.

In total, 199 articles were extracted from the database search; 110 were identified after eliminating duplicates. Primary screening resulted in the exclusion of 75 articles. After secondary screening of the remaining 35 articles, 19 articles that met the eligibility criteria were selected. The reference lists of the accepted articles were also checked, and primary and secondary screenings were conducted again for the relevant literature; 2 additional articles meeting the eligibility criteria were identified. Finally, 21 articles (15–35) were selected for this scoping review (Fig. 1).

### Study characteristics

The included studies were conducted in 10 countries: the United States, Japan and Korea (4 articles each), Turkey and Brazil (2 articles), and the Czech Republic, China, Sweden, Hong Kong, and Taiwan (1 article each). Many of the studies (16 articles) had a sample size of < 100 (*n* = 27–99), 4 articles had a sample size between 100 and 200, and only 1 article had a sample size of > 200 (Table I). In terms of fall occurrences, 6 studies used inpatient fall records, 3 studies recorded falls prospectively, and 11 studies used a history of falls (Table II). Thirteen studies identified 1 or more falls as a fall group, 5 identified 2 or more falls, and 2 analysed both. The percentage of fallers ranged from 13% to 61%. The AUCs ranged from 0.61 to 0.92, and many studies reported cutoff values associated with AUCs below 0.7 (Table II). Assessments and cutoff values with an AUC of 0.7 or higher, which is generally considered an acceptable level (36) and clinically useful, are tabulated in Table SI.

**Table I:** List of articles reviewed

Author (year)	Country	Post-stroke stage	Sample size	Main selection criteria of participants		
				Stroke type	Physical function	Cognitive function
Alenazi et al. (2018) (15)	United States	Chronic	181		The ability to walk > 10 m without an orthotic device	MMSE ≥ 24
Alzayer et al. (2009) (16)	United States	Chronic	44		The ability to walk 10 m independently with or without an assistive device	The ability to follow 3-step commands
An et al. (2014) (17)	Korea	Chronic	72		The ability to walk more than 10 m without a walking aid	MMSE ≥ 24
An et al. (2017) (18)	Korea	Chronic	57		The ability to walk more than 10 m without a walking aid	MMSE > 24
Belgen et al. (2006) (19)	United States	Chronic	50		The ability to walk 10 m with no physical assistance with or without any assistive device	The ability to follow 3-step commands
Beninato et al. (2009) (20)	United States	Chronic	27		The ability to ambulate independently at least 10 m with or without an assistive device	The ability to follow 3-step commands
Faria-Fortini et al. (2021) (21)	Brazil	Chronic	105	Primary or recurrent unilateral stroke	The ability to walk 10 m with or without an assistive device	Except for MMSE < 13 or 18 or 26 (education-adjusted cutoff scores)
Fiedorová et al. (2022) (22)	Czech Republic	Subacute	84	Primary ischaemic stroke	FAC 3–5, and the ability to stand without support for 5 min	Except for severe phatic disorder
Huo et al. (2009) (23)	China	Chronic	27		The ability to walk independently with or without a cane	Except for higher cortical dysfunction or severe impair-ment of speech
Kızılkaya et al. (2023) (24)	Turkey	Chronic	39	First unilateral anterior circulation stroke	The ability to walk at least 10 m without assistance, and BRS 3–6	MMSE ≥ 24
Lee et al. (2021) (25)	Korea	Unclear	227		FAC > 2	
Maeda et al. (2009) (26)	Japan	Chronic	72			
Park et al. (2018) (27)	Korea	Chronic	99			Except for MMSE-K < 18
Persson et al. (2011) (28)	Sweden	Acute	96	First-ever stroke		Except for diagnosis of dementia or severe psychiatric diseases
Pinto et al. (2014) (29)	Brazil	Chronic	150	Ischaemic or haemorrhagic stroke		The ability to understand the tests
Sahin et al. (2019) (30)	Turkey	Chronic	50		The ability to stand for 2 min unassisted, and walk unassisted or assisted (with cane) 6 m	MMSE ≥ 24
Takatori et al. (2009) (31)	Japan	Subacute, chronic	60		The ability to stand unassisted for at least 1 min	MMSE ≥ 24, and no severe higher brain function disorders
Takatori et al. (2009) (32)	Japan	Subacute, chronic	76		The ability to stand unassisted for at least 1 min	MMSE ≥ 24, and no severe higher brain function disorders
Tsang et al. (2013) (33)	Hong Kong	Chronic	106			The ability to understand verbal instructions
Yamasaki et al. (2023) (34)	Japan	Subacute	33	First stroke involving the vertebrobasilar territory	Presence of ataxic symptoms in one upper or lower limb, and BRS ≥ 5	No cognitive impairment, or higher brain dysfunction
Zou et al. (2021) (35)	Taiwan	Chronic	30	Single and unilateral stroke	The ability to walk independently over a distance of 10 m without walking aids or orthoses	No cognitive impairments or aphasia

MMSE: Mini-Mental State Examination; FAC: Functional Ambulation Categories; BRS: Brunnstrom Recovery Stage; MMSE-K: Mini-Mental State Examination-Korean version.

**Table II:** Cutoff values and accuracy for predicting falls

Author (year)	Fall monitoring		Falling groups	Predictor	Cutoff	AUC	Sens	Spec
	Period	Setting						
Alenazi et al. (15)	42 weeks	Community	≥ 2 falls (*n* = 24) vs 1 or no fall (*n* = 157)	FRT	18.15	0.66	76%	56%
				PHQ-9	2.5	0.62	60%	65%
Alzayer et al. (16)	Last 6 months	Community	≥ 2 falls (*n* = 10) vs 1 or no fall (*n* = 34)	BBS	52	0.67	90%	41%
An et al. (17)	Last 12 months	Unclear	≥ 2 falls (*n* = 44) vs non-faller (*n* = 28)	POMA	12.5	0.78	72%	74%
An et al. (18)	Last 6 months	Unclear	Faller (*n* = 25) vs non-faller (*n* = 32)	DGI-4	9.5	0.77	68%	59%
				DGI-8	16.5	0.78	60%	72%
Belgen et al. (19)	Last 6 months	Community	≥ 2 falls (*n* = 11) vs 1 or no fall (*n* = 39)	BBS	52	0.72	91%	42%
			Faller (*n* = 20) vs non-faller (*n* = 30)	Swedish FES	17.5	0.71	90%	53%
Beninato et al. (20)	Last 6 months	Community	≥ 2 falls (*n* = 9) vs 1 or no fall (*n* = 18)	ABC Scale	81.1	0.92	100%	72%
				BBS	49	0.76	78%	72%
				SIS-16	61.7	0.86	78%	89%
				STS	17.9	0.66	67%	72%
Faria-Fortini et al. (21)	Last 6 months	Community	Faller (*n* = 42) vs non-faller (*n* = 63)	FES-International	28	0.71	71%	57%
Fiedorová et al. (22)	6 months	Community	Faller (*n* = 32) vs non-faller (*n* = 52)	BBS	35	0.66	44%	89%
				BBS	42	0.62	56%	67%
				FES-International	27	0.69	81%	56%
				FES-International	29	0.68	72%	64%
				SOT	60	0.69	72%	65%
Huo et al. (23)	Last 12 months	Unclear	Faller (*n* = 7) vs no-faller (*n* = 20)	P-RT	626	0.77	86%	70%
Kızılkaya et al. (24)	Last 6 months	Unclear	Unclear	Turkish FAB	21.5	0.75	84%	61%
Lee et al. (25)	During hospital stay	Hospital	Faller (*n* = 44) vs non-faller (*n* = 183)	MFS	32.5	0.61	79%	38%
				TUG	18.58	0.69	78%	55%
Maeda et al. (26)	During hospital stay	Hospital	Faller (*n* = 27) vs non-faller (*n* = 45)	BBS	29	0.81	80%	78%
Park et al. (27)	Unclear	Community	Faller (*n* = 35) vs non-faller (*n* = 64)	ABC Scale	63.75	0.69	41%	92%
				Korean FES	66.5	0.68	70%	64%
Persson et al. (28)	12 months	Unclear	Faller (*n* = 46) vs non-faller (*n* = 50)	10MWT	12	0.74	80%	58%
				BBS	42	0.69	69%	65%
				M-MAS UAS-95	50	0.72	74%	58%
				SwePASS	32	0.73	82%	50%
				TUG	15	0.7	63%	58%
Pinto et al. (29)	Last 12 months	Community	Faller (*n* = 56) vs non-faller (*n* = 94)	TUG	25	0.66	36%	90%
Sahin et al. (30)	Last 12 months	Unclear	Faller (*n* = 26) vs non-faller (*n* = 24)	ABC Scale	55.31	0.78	75%	81%
				BBS	46.5	0.81	75%	77%
				BESTest	69.44	0.84	75%	85%
Takatori et al. (31)	3 months	Hospital	≥ 2 falls (*n* = 15) vs 1 or no fall (*n* = 45)	EED	6.3	0.8	80%	78%
Takatori et al. (32)	5 months	Hospital	Faller (*n* = 37) vs non-faller (*n* = 39)	EED	6.1	0.7	69%	82%
			≥ 2 falls (*n* = 21) vs 1 or no fall (*n* = 55)	EED	6.3	0.8	81%	78%
Tsang et al. (33)	Last 12 months	Community	Faller (*n* = 25) vs non-faller (*n* = 81)	BBS	50.5	0.72	52%	80%
				FRT	24.1	0.67	52%	74%
				Mini-BESTest	17.5	0.64	64%	64%
				OLS: nonparetic side	3.6	0.64	40%	84%
				OLS: paretic side	0.9	0.67	56%	78%
				TUG	19	0.66	61%	67%
Yamasaki et al. (34)	1 month	Hospital	Faller (*n* = 10) vs non-faller (*n* = 23)	STV	6.35	0.84	80%	74%
Zou et al. (35)	12 months	Hospital	Faller (*n* = 9) vs non-faller (*n* = 21)	Turn duration	4	0.75	67%	80%
				Turn step	7	0.73	56%	85%

AUC: area under the curve; Sens: sensitivity; Spec: specificity; FRT: Functional Reach Test; PHQ-9: Patient Health Questionnaire-9; BBS: Berg Balance Scale; POMA: Performance-Oriented Mobility Assessment; DGI: Dynamic Gait Index; FES: Falls Efficacy Scale; ABC Scale: Activities-specific Balance Confidence Scale; SIS: Stroke Impact Scale; STS: Five Times Sit to Stand Test; SOT: Sensory Organization Test; P-RT: Probe Reaction Time; FAB: Fullerton Advanced Balance Scale; MFS: Morse Fall Scale; TUG: Timed Up and Go; 10MWT: 10 Meter Walk Test; M-MAS UAS-95: Modified Motor Assessment Scale Uppsala Akademiska Sjukhus; SwePASS: Swedish version of the Postural Assessment Scale for Stroke patients; BESTest: Balance Evaluation Systems Test; EED: error in estimated distance; OLS: one-leg standing; STV: stride time variability; FES: Falls Efficacy Scale.

### Study design and outcome characteristics

Most studies used gait- and balance-related assessments to identify patients at risk of falls (Table II). Six studies used confidence not to fall assessments (Activities-specific Balance Confidence [ABC] Scale, Falls Efficacy Scale [FES]) (19–22, 27, 30); 1 study used a comprehensive assessment (Morse Fall Scale [MFS]) (25), and 1 used depression (9-item Patient Health Questionnaire) as the measure of fall risk (15). The most used individual indicator was the Berg Balance Scale (BBS; 8 studies) (16, 19, 20, 22, 26, 28, 30, 33), followed by the TUG (4 studies) (25, 28, 29, 33), FES-related indicators (4 studies) (19, 21, 22, 27), and ABC Scale (3 studies) (20, 27, 30). None of the articles included in this review calculated a cutoff value to identify the risk of falling based on a measure of cognitive function.

**Fig. 2:**
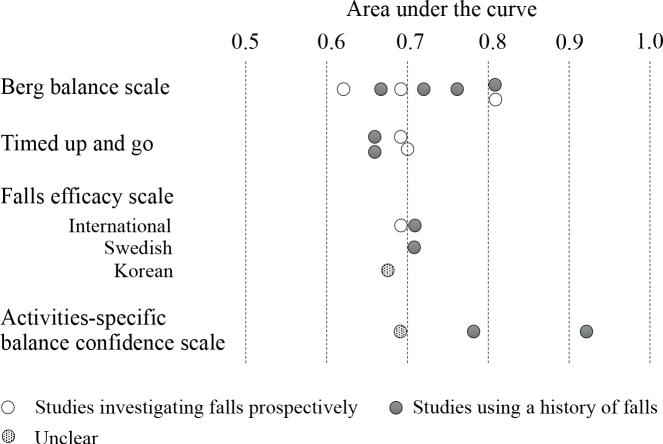
Distribution of area under the curves for indicators used in 3 or more articles.

Integrating the information from the 6 studies that had BBS cutoff sensitivity and specificity values greater than 50% revealed that the cutoff and AUC values varied widely, ranging from 29 to 50.5 points and 0.62 to 0.81, respectively (Fig. 2). However, the cutoff values used in the 3 studies (20, 30, 33) that applied BBS for community-dwelling individuals with chronic stroke were relatively consistent, ranging from 46.5 to 50.5 points (AUC 0.72–0.81). In the only study examining falls during hospitalization (26), cutoff values tended to be low, at 29 points (AUC 0.81).

The TUG test (4 out of 21 studies) was the next most commonly used individual indicator. The cutoff values and AUCs in the 3 studies (25, 28, 33) with TUG sensitivity and specificity cutoff values greater than 50% ranged from 15 to 19 s and 0.66 to 0.7, respectively, with relatively consistent results.

FES-related assessments were used in 4 studies (19, 21, 22, 27); however, the versions used varied: 1 Korean (27), 1 Swedish (19), and 2 international versions (21, 22). The cutoff values and AUCs in the 2 studies (21, 22) using the FES-International (FES-I) were consistent, ranging from 27 to 29 and 0.68 to 0.71, respectively. The 3 studies (20, 27, 30) using the ABC Scale had a high variability of cutoff values and AUCs, ranging from 55.3 to 81.1 and 0.69 to 0.92, respectively. The highest AUC among the 21 studies was reported by Beninato et al. (20), who used the ABC Scale to identify community-dwelling individuals at risk of falling after stroke (AUC 0.92, sensitivity 100%, specificity 72%); however, the study had a small sample size of 27 and the study design was not prospective, as it investigated past falls.

## DISCUSSION

This scoping review summarizes the cutoff values reported to date used to identify fall risk after stroke and examines their commonalities and limitations. Al-though the studies included in this review were conducted under various conditions using numerous measures, the range of AUCs varied from 0.61 to 0.92, with many results below 0.7, indicating certain limitations when using a single cutoff value. An existing review (11) has reported AUCs of 0.62–0.87 when using multivariate prediction models, indicating that the identification of these individuals remains a challenge.

The BBS was found to be an indicator with relatively high discriminatory power to differentiate between fall and non-fall groups in community-dwelling individuals with chronic stroke, although it was difficult to integrate the results owing to the varying conditions among the studies. From 3 different studies, the cutoff values were relatively consistent, ranging from 46.5 to 50.5 points, and the AUCs ranged from 0.72 to 0.81, above the level generally considered acceptable (AUC > 0.7) (36). Existing reviews of studies on older adults have reported BBS cutoff values of 45–51 (37) or 46–54 (38) points for identifying fall risk in older adults. The present review revealed that the BBS cutoff values were comparable among studies of community-dwelling individuals with chronic stroke. Our results also indicate that BBS cutoff values tended to be lower (29 points) when identifying inpatient fall risk. Although this finding is based on only 1 study and has limited generalizability at this stage, it is a reasonable result considering that inpatients with concern about fall are provided with the necessary assistance and monitoring whenever possible during walking.

In contrast, the primary cut-off range for the TUG identified in this study – 15–19 s – was slower than the cut-off value of 12.3 s established in previous studies for community-dwelling older adults (13, 39). Previous studies have reported that individuals with chronic stroke typically take longer to complete the TUG compared with community-dwelling older adults (40). Therefore, rehabilitation therapists should recognize that the optimal TUG cut-off values for predicting falls in older adults may not be applicable to individuals with stroke. In addition, for TUG and FES-I, the cutoff values were relatively consistent across the literature, but the AUCs were low (0.66–0.7 and 0.68–0.71, respectively), suggesting limited accuracy. Several reviews of community-dwelling older adults have noted limitations in identifying individuals at high risk of falls using TUG (41, 42). The results of this review reveal that caution should be exercised in relying solely on the cutoff values of the FES-I as well as the TUG to determine the risk of falls. Therefore, we advocate for the combined use of TUG and FES-I cutoff values alongside other indicators for fall risk.

The highest AUC in the articles used in this review was 0.92, and the indicator was the ABC scale. The results indicate a close association between falls and confidence in balance during activities. However, it is important to note that all of the studies using the ABC scale (20, 27, 30) in this review examined past falls, which is important for proper interpretation. In other words, the results of previous studies may indicate that stroke patients lose confidence in their balance due to falls. In particular, the report by Beninato et al., (20), which reported the highest AUC, was based on the multiple-fall group, who experienced 2 or more falls. It may not be surprising that multiple falls are strongly associated with reduced balance confidence. Therefore, further prospectively designed studies are needed to determine whether the cutoff value of the ABC scale can be used as a predictor of falls.

Notably, we found that the cutoff values of cognitive function for identifying fall risk are unclear. In this review, some studies used the level of cognitive function as a selection criterion for individuals; however, the number of studies was small, and it was difficult to integrate the information. However, the 2010 review by Campbell & Matthews (3) noted a poorly established role of cognitive function in falls in patients after stroke. To our best knowledge, only 1 study has used the Montreal cognitive assessment to predict falls during hospital stays in acute care hospitals (43), which was not included in this review. This is a potential gap in the literature that warrants further study.

### Strength and limitations

An important finding of the present review was the certain degree of reliability and accuracy of BBS (46.5–50.5 points, AUC 0.72–0.81) in identifying fall risk among community-dwelling individuals with chronic stroke. However, a limitation of this review was that it was limited to studies in which the AUC, sensitivity, and specificity were calculated to ascertain accuracy. No searches were conducted in electronic databases other than PubMed, CINAHL, and Scopus. Therefore, our review did not include all studies that calculated the cutoff values for predicting the risk of falls. Moreover, our scoping review did not include a quality assessment of each study, which is standard practice in systematic reviews.

### Conclusion

This review is the first to summarize the cutoff values for identifying the risk of falls in patients after stroke, identifying certain limits of accuracy and contributing to the appropriate use of cutoff values in clinical practice.

Among various assessments, the BBS has the best-studied cutoff values associated with falls and is a reliable indicator for detecting fall risk, especially in community-dwelling individuals with chronic stroke. Overall, however, the accuracy of single cutoff values, including TUG and FES-I, in predicting falls in stroke patients remains uncertain and therapists should be aware of the limitations of accuracy. In addition, many indicators are insufficiently validated, including cognitive functions, necessitating further research.

## Supplementary Material

RELATIVE AEROBIC LOAD OF WALKING IN PEOPLE WITH MULTIPLE SCLEROSIS
