# Supplementary material for: SCREENING CUTOFF VALUES TO IDENTIFY THE RISK OF FALLS AFTER STROKE: A SCOPING REVIEW
Source: J Rehabil Med. 2024 Oct 24;56:40560. doi: 10.2340/jrm.v56.40560 (PMC11520422; doi:10.2340/jrm.v56.40560)
Supplement: RELATIVE AEROBIC LOAD OF WALKING IN PEOPLE WITH MULTIPLE SCLEROSIS [file JRM-56-40560-s1.pdf]

Supplementary material has been published as submitted. It has not been copyedited, or typeset by Journal of Rehabilitation Medicine

Table SI. List of fall-related indicators with AUC of 0.7 or higher and its cutoff values

Author (year)	Post-stroke	Main selection criteria of participants	Fall monitoring		Predictor	Cutoff	AUC	Sensitivity	Specificity
			Period	Setting					
An et al. (2014)	Chronic	The ability to walk more than 10 m without a walking aid & MMSE ≥24	Last 12 months	Unclear	POMA	12.5	0.78	72%	74%
An et al. (2017)	Chronic	The ability to walk more than 10 m without a walking aid & MMSE >24	Last 6 months	Unclear	DGI-4	9.5	0.77	68%	59%
					DGI-8	16.5	0.78	60%	72%
Belgen et al. (2006)	Chronic	The ability to walk 10 m with no physical assistance with or without any assistive device & the ability to follow 3-step commands	Last 6 months	Community	BBS	52	0.72	91%	42%
					Swedish FES	17.5	0.71	90%	53%
Beninato et al. (2009)	Chronic	The ability to ambulate independently at least 10 m with or without an assistive device & the ability to follow 3-step commands	Last 6 months	Community	ABC Scale	81.1	0.92	100%	72%
					BBS	49	0.76	78%	72%
					SIS-16	61.7	0.86	78%	89%
Faria-Fortini et al. (2021)	Chronic	The ability to walk 10 m with or without an assistive device<br>Except for MMSE <13 or 18 or 26 (education-adjusted cutoff scores)	Last 6 months	Community	FES-International	28	0.71	71%	57%
Huo et al. (2009)	Chronic	The ability to walk independently with or without a cane<br>Except for higher cortical dysfunction or severe impairment of speech	Last 12 months	Unclear	P-RT	626	0.77	86%	70%
Kızılkaya et al. (2023)	Chronic	First unilateral anterior circulation stroke & MMSE ≥24<br>The ability to walk at least 10 m without assistance, and BRS 3–6	Last 6 months	Unclear	Turkish FAB	21.5	0.75	84%	61%

Maeda et al. (2009)	Chronic		During hospital stay	Hospital	BBS	29	0.81	80%	78%
Persson et al. (2011)	Acute	First-ever stroke & except for diagnosis of dementia or severe psychiatric diseases	12 months	Unclear	10MWT	12	0.74	80%	58%
					M-MAS UAS-95	50	0.72	74%	58%
					SwePASS	32	0.73	82%	50%
					TUG	15	0.7	63%	58%
Sahin et al. (2019)	Chronic	The ability to stand for 2 min unassisted, and walk unassisted or assisted (with cane) 6 m & MMSE ≥24	Last 12 months	Unclear	ABC Scale	55.31	0.78	75%	81%
					BBS	46.5	0.81	75%	77%
					BESTest	69.44	0.84	75%	85%
Takatori et al. (2009)	Subacute, chronic	The ability to stand unassisted for at least 1 min<br>MMSE ≥24, and no severe higher brain function disorders	3 months	Hospital	EED	6.3	0.8	80%	78%
Takatori et al. (2009)	Subacute, chronic	The ability to stand unassisted for at least 1 min<br>MMSE ≥24, and no severe higher brain function disorders	5 months	Hospital	EED	6.1	0.7	69%	82%
					EED	6.3	0.8	81%	78%
Tsang et al. (2013)	Chronic	The ability to understand verbal instructions	Last 12 months	Community	BBS	50.5	0.72	52%	80%
Yamasaki et al. (2023)	Subacute	First stroke involving the vertebrobasilar territory	1 month	Hospital	STV	6.35	0.84	80%	74%
Zou et al. (2021)	Chronic	Single and unilateral stroke	12 months	Hospital	Turn duration	4	0.75	67%	80%
					Turn step	7	0.73	56%	85%

AUC, area under the curve; MMSE, Mini-Mental State Examination; POMA, Performance-Oriented Mobility Assessment; DGI, Dynamic Gait Index; BBS, Berg Balance Scale; FES, Falls Efficacy Scale; ABC Scale; Activities-specific Balance Confidence Scale; SIS, Stroke Impact Scale; P-RT, Probe Reaction Time; BRS, Brunnstrom Recovery Stage; FAB, Fullerton Advanced Balance Scale; 10MWT, 10 Meter Walk Test; M-MAS UAS-95, Modified Motor Assessment Scale Uppsala Akademiska Sjukhus; SwePASS, Swedish version of the Postural Assessment Scale for Stroke patients; TUG, Timed Up and Go; BESTest, Balance Evaluation Systems Test; EED, error in estimated distance; STV, stride time variability
